# ENHANCING DEMENTIA CARE PARTNER ENGAGEMENT IN FALL PREVENTION PROGRAMS: A SYSTEMATIC REVIEW

**DOI:** 10.1093/geroni/igae098.3364

**Published:** 2024-12-31

**Authors:** Xiaoyi Zeng, Yuanjin Zhou, Namkee Choi

**Affiliations:** The University of Texas Austin, Austin, Texas, United States; The University of Texas Austin, Austin, Texas, United States; The University of Texas Austin, Austin, Texas, United States

## Abstract

Community-dwelling older people with cognitive impairment/dementia (CI/D) experience a higher risk of falls than age-matched peers without CI/D. Fall prevention programs for this population often engage their family/friend care partners. Care partner engagement (CPE) has five components: care partners’ recruitment, retention, attendance, active participation, and maintenance of changes. Little is known about the facilitators and barriers that impact CPE in those programs and strategies used to enhance CPE. We conducted a mixed-methods systematic review following PRISMA guidelines (PROSPERO: CRD42023422200). Twenty-nine unique fall prevention programs from 32 studies were included. Most studies described retention (n=19, 60%), attendance (n=12, 38%), and active participation (n=32, 100%) aspects of CPE. Fewer studies illustrated factors related to care partner recruitment (n=6, 19%) and maintenance of change (n=6, 19%). Commonly reported CPE facilitators were older people with CI/D and care partners’ perceived benefits and suitableness of the fall prevention programs and positive relationships between the dyads. The main barriers to CPE are the dyads’ poor health and the burden related to the study logistics of participating in the programs. Program characteristics (e.g., structured/group format, social engagement opportunities, delivery mechanisms, timing) and providers’ characteristics are commonly reported organizational-level factors impacting CPE. Twenty-seven studies mentioned the use of educational (e.g., training, consultation, booklet), relational (e.g., strengthening care relationships, engaging paid caregivers), and behavioral strategies (e.g., action plans, visual reminders, cognitive approach, tracking logs) to promote CPE. Findings proposed a theoretical framework and identified potential strategies to enhance CPE in fall prevention programs for older people with CI/D.

